# Coping With COVID-19: Learning From Past Pandemics to Avoid Pitfalls and Panic

**DOI:** 10.9745/GHSP-D-20-00189

**Published:** 2020-06-30

**Authors:** Daniel T. Halperin

**Affiliations:** a Gillings School of Global Public Health, University of North Carolina, Chapel Hill, NC, USA.

## Abstract

It is imperative to concur on the main transmission routes of COVID-19 to explain risk and determine the most effective means to reduce illness and mortality. We must avoid generating irrational fear and maintain a broader perspective in the pandemic response, including assessing the possibility for substantial unintended consequences.

## Key Messages

- As we wrestle with how best to mitigate COVID-19, it is imperative to concur on the likely main drivers of transmission (notably, infection clusters resulting from prolonged indoor respiratory exposure) in order to clearly explain risk and to determine the most effective, realistic behavioral and other means to reduce illness and mortality.
- At the same time, we must avoid generating irrational fear and maintain a broader perspective, including assessing the possibility for substantial unintended consequences from the response to the pandemic.

## UNCANNY SIMILARITIES WITH THE LAST MAJOR PANDEMIC

In June 1981, when the first cases were reported of what became known as AIDS, I was living in the San Francisco Bay area. As the waves of death mounted, I volunteered at a hospice in Oakland, California, and later conducted epidemiological research at the University of California.

There are major differences between HIV and severe acute respiratory syndrome coronavirus 2 (SARS-CoV-2) and their resulting pandemics (AIDS and coronavirus disease ([COVID-19]). However, I’m having déjà vu: from the devastating number of deaths and the pervasive atmosphere of confusion, fear, and often panic.

Tragically, political leaders from Ronald Reagan to Nelson Mandela were slow to respond to the AIDS epidemic. All sides engaged in acrimonious ideological warfare that often ignored the epidemiological evidence. In hindsight, health authorities also made some decisions—especially under the pressure of needing to act immediately—that led to suboptimal and ultimately costly outcomes.^1^^,^^2^ Policies often became hardwired over time and difficult to walk back, even after new evidence appeared. Well-meaning but overly simplistic messages such as, “always use a condom with anyone or die” inadvertently created other problems.^1^^,^^3^^,^^4^ Earlier openness to innovative approaches, such as male circumcision and addressing sexual networks, could have saved many lives, particularly in sub-Saharan Africa.^1^^,^^2^^,^^5^ In subsequent years, as greater funding for research and treatment eventually poured in, a kind of “AIDS exceptionalism” also became imbedded.^6^

During the first years of the AIDS response, much was unknown regarding the causes and main modes of transmission. Yet, even after HIV was identified in 1983 and the basic science became clearer, a great deal of uncertainty, persecution of marginalized groups, and terror persisted. Rumors proliferated that anything from mosquitoes to using contaminated condoms to sharing toothbrushes were spreading the virus. In the 1990s, after Earvin “Magic” Johnson tested positive for HIV, counseling centers became overrun by the “worried well.”^1^^,^^3^ Heterosexual college students flooded centers to get tested, petrified from having engaged in deep kissing or intimate touching “without protection,” thereby diverting attention from those who were actually at significant risk of infection.

## CONFUSION AND PANIC RETURNS WITH THIS PANDEMIC

With COVID-19, much remains unclear, but some basic facts are known and more emerge daily. Yet, a palpable climate of confusion and anxiety pervades. (One mind-boggling indication is that the Johns Hopkins University Coronavirus Resource Center website is recording some 4 billion hits a day!^7^) Under such circumstances, fear is understandable and can help motivate behavior change.^1^^,^^8^ However, when fear becomes irrational or leads to panic, it often results in poor decision making and other unintended consequences.^9^^,^^10^ Moreover, there are troubling signs that we have failed to learn other important lessons from the previous pandemic, including the danger of polarized infighting. For example, politicians and the media as well as some medical experts are presenting us with a false dichotomy: having to choose between recklessly abandoning mitigation efforts to reopen the economy versus rigidly continuing present lockdown measures.

The U.S. territory of Puerto Rico where I reside implemented a nearly complete shutdown in mid-March after the first death here (of an Italian cruise ship passenger). Since then, many people, convinced the virus is “everywhere” and infection is nearly unavoidable, won’t leave their homes even to pick up groceries. When delivery services became overwhelmed, elderly and sick persons sometimes have not been able to obtain essential supplies. Most of those who do drive or go outdoors use masks (needlessly) even when far away from other people. Wearing masks in the hot, humid climate can be uncomfortable and has created issues, including elderly persons fainting while waiting in the sun for a long time to enter stores (which often only allow a handful of customers to enter at once). Until recently, joggers and others were stopped and occasionally fined by police for venturing outside or for violating the 7 pm curfew, which remains in effect after nearly 3 months. (Even in most of the world’s hardest-hit countries, such as Spain and Italy, people are now allowed outdoors to exercise.) Numerous restaurants, especially Asian-owned ones, have closed after losing takeout customers. Some stores require customers to wear gloves, despite evidence suggesting limited utility or that their use may actually increase risk of infection.^11^

## WHAT ARE THE MAIN RISK FACTORS FOR SEVERE OUTCOMES?

In Puerto Rico, as in other parts of the world, many people (even many youth) with asthma are terrified of experiencing severe outcomes if they become infected with the virus, prompting shortages of inhalers and other critical supplies. The U.S. Centers for Disease and Prevention (CDC) website lists people with asthma near the top of those at risk of severe COVID-19 outcomes, even though only 1 clinical study has investigated whether a relationship exists and has found no link.^12^ Although other emerging data strongly appear to confirm the lack of an association,^13^ it is unclear whether the CDC will correct its public information.

What is clear, based on evidence from several countries (and despite media attention to statistically anomalous cases of healthy and younger victims), is that severe outcomes and deaths from COVID-19 are overwhelmingly associated with preexisting (and especially multiple) serious illnesses such as diabetes and heart disease,^14^^–^^16^ more so in men and particularly when exacerbated by obesity and smoking.^17^^,^^18^

Indeed, it may be that advanced age alone, in the absence of such predisposing conditions, is less of an independent risk factor than has been assumed. Firstly, the elderly are more likely to have chronic illnesses, which confounds the association between outcomes and age. Moreover, the fact that between 96% (in the United States^16^) and more than 99% (in Italy^14^) of COVID-19-related deaths, at any age, have occurred in persons with preexisting conditions could suggest that even very old but otherwise healthy people may not be at greatly elevated risk of dying from the disease. Further research and analysis, including assessing whether the important angiotensin-converting enzyme 2 protein (ACE-2) is more prevalent in the elderly^19^ could help explain the often higher infection (not only higher mortality) rates in older populations.^20^ In any case, such data underscore the ongoing need in general to prioritize preventing chronic diseases, which kill more than 40 million people annually (over 80% in lower- and middle-income countries),^21^ and to address underlying conditions such as obesity and smoking.^22^^,^^23^

## CLEAR EVIDENCE-BASED INFORMATION IS OFTEN LACKING

Regarding COVID-19 prevention, it is imperative for experts to agree on what are the likely main transmission routes and to carefully determine which are, accordingly, the most effective (and realistically achievable) behavioral and other ways to reduce morbidity and mortality. It is probable that, as with other *respiratory* illnesses such as influenza, most COVID-19 infections occur from close exposure to coughing, sneezing, shouting, singing, or other direct and *relatively prolonged contact* with someone who is symptomatic or presymptomatic. (There is evidence that some asymptomatic carriers are contagious, but from existing studies they appear not to represent a very substantial proportion of total COVID-19 transmission.)

It is probable that most infections occur from close exposure to coughing, sneezing, shouting, singing, or other direct and prolonged contact with an infected person.

In February, a team of World Health Organization (WHO) researchers led by David Heymann investigated the outbreak in Wuhan, China, and concluded that the large majority of transmission events occurred within *indoor clusters*^24^ between family members (accounting for 75%–85% of estimated infections) and coworkers, with no identified cases of child-to-adult transmission identified.^15^ In addition, some data suggest that severity of outcomes is associated with initial exposure viral-load levels.^24^^–^^26^ Moreover, it increasingly appears that infection risk from contaminated surfaces has been at least somewhat overstated, as the CDC recently acknowledged.^27^ Indeed, it is conceivable that future science historians may conclude that many current COVID-19 prevention strategies had little if any impact, particularly because they targeted drivers of spread accounting for no more than a small proportion of total infections.

Future historians may conclude that many current prevention strategies had little impact because they targeted unlikely drivers of infection.

## HIGH RISK, LOW RISK, OR NO RISK OF TRANSMISSION

As some experts eventually did with HIV,^1^^,^^4^ they could also help the public distinguish between those behaviors and situations posing the highest risk for COVID-19 infection, those of likely lower risk (such as the virus lingering on hard surfaces for extended periods), and those of highly unlikely or no risk (such as being outdoors with no one else around). Although the CDC has posted some basic guidance on its website (in the Frequently Asked Questions section) regarding how COVID-19 is mainly transmitted, the public would benefit from a more clearly communicated^28^ and much more robust public information campaign (e.g., including the virtual equivalent of placing a leaflet under every U.S. resident’s door). This would help reduce time and attention spent addressing low-risk concerns, such as when healthy people avoid leaving home for necessary activities even if carefully taking precautions.

There is a crucial distinction between risk of indoor transmission—where physical distancing (whether mandated or voluntary) and perhaps other measures^28^^,^^29^ are critical—versus risk of outdoor transmission, which is far lower (possibly by an order of magnitude) for various reasons, including dissipation of droplets in the air^28^^,^^30^ and the deactivating effects of ultraviolet radiation and heat.^31^^–^^33^ A contact tracing study from China found that 80% of infections involved household members and 34% involved mass transit (multiple potential transmission routes were considered), whereas only a single infection event of the 7,324 cases investigated was linked to casual outdoor transmission.^28^^,^^30^^,^^34^

There is a crucial distinction between risk of indoor transmission, where physical distancing is critical, versus the risk of outdoor transmission, which is far lower.

Although politicians and the media have been obsessed with the danger of frolicking on beaches (or of participating in protest gatherings), a vastly greater risk is the common (public health) admonition for sick persons to remain home as long as possible before seeking hospital care, without providing access to alternative, clinically-provisioned quarantine residences, as several Asian countries and Iceland have successfully instituted.^35^^–^^37^ Delays in seeking care not only diminish survival chances but also expose household members to significant infection risk.^14^^,^^35^^,^^36^^,^^38^

## IS 6 FEET DISTANCING STRICTLY NECESSARY?

One example of inconsistent public health messaging is that European and Asian authorities and the WHO recommended physical distancing based on data that droplets containing the virus had been identified almost a meter away from coughing individuals. In the United States, for some reason 1 meter was initially translated into 5 feet and subsequently became “over 6 feet.” Although perhaps arguably not the highest priority, it would be useful for the CDC and other experts to determine whether such abundance-of-caution guidance is worth maintaining or perhaps is not scientifically warranted, and may inadvertently feed excessive concern. (In fact, the entire concept of physical or “social” distancing is not specifically relevant to transmission risk, primarily related to respiratory droplets: the pertinent issue is not the distance per se between people's bodies but rather between their faces, particularly if unmasked. For example, if 2 people are positioned back-to-back, then obviously the distance can safely be much less.)

This issue of distancing is particularly relevant as weather improves and outdoor exercise becomes more common, as many health departments^28^^,^^30^^,^^39^ encourage people to do (even though a hypothetical model based on untested assumptions sparked alarm by suggesting that joggers or cyclists could spread the virus over greater distances^40^). And critically, as the economy begins to reopen, it would be especially challenging for some businesses (and eventually schools) to adhere strictly to a 6-foot rule. This could be particularly excessive for outdoor activities, including construction, farming, recreation, and outdoor dining. It is certainly more practical to maintain a distance of about 3 feet than 6 feet in many situations, such as grocery shopping (where interactions are typically brief) or while strolling with a companion.

The pertinent factor is not the physical distance between people's bodies, but rather the distance between their faces.

## COVID-19 AND CHILDREN: MUST SCHOOLS REMAIN CLOSED?

Indeed, it is likely that more “surgical”—more carefully targeted and realistic, evidence-based approaches^4^^,^^41–^^43^—could be similarly efficacious as more extreme isolation strategies that have been widely implemented. For example, Singapore had initially achieved a notably effective response without shutting schools.^44^ (However, subsequently there was a surge in cases due to an outbreak in crowded migrant-worker dormitories.) Taiwan, which never closed its schools, has continued to report very few cases.

Similar to the first severe acute respiratory syndrome (SARS) epidemic in 2002–2004,^45^^,^^46^ the vast majority of children infected with SARS-CoV-2 escape severe outcomes. There has been much media attention to multisystem inflammatory syndrome in children (MIS-C), which has features similar to Kawasaki disease. However, of the more than 7 million COVID-19 infections reported worldwide to date, only a few hundred cases of MIS-C have been identified so far.^47^^,^^48^ (Although the usual Kawasaki disease is more common in East Asia, in the United States about 5,000 cases occur annually.^48^) Of more than 400,000 COVID-19 deaths reported worldwide, some 20 children are known to have died, about half of them in the United States and the rest in Europe. By comparison, more than 200 children died last year from the flu in the United States alone, along with some 10,000 others from various childhood diseases.^49^ Further contextualizing the MIS-C and other childhood deaths from COVID-19, in the United States, per-capita mortality in persons aged 85 years and older is 2,000 times higher than in children aged 15 years and younger.^50^ (An intriguing question posed by some researchers is whether MIS-C is definitely or always caused by COVID-19, considering that in some cases up to one-third of afflicted children have tested negative for COVID-19, both on polymerase chain reaction and antibody tests.^51^) Although the emerging MIS-C must be closely monitored, as with Kawasaki disease most cases appear to recover fairly rapidly, especially if detected and treated early.^46^^–^^48^

Because young people typically come in contact with many other children and adults, they are often efficient spreaders of respiratory pathogens. However, growing evidence suggests that, as with the earlier SARS,^45^^,^^46^ children are less likely to become infected with SARS-CoV-2.^52^^–^^58^ According to the CDC, only about 1.5% of U.S. cases of COVID-19 have been reported in persons aged 18 years or younger.^59^ Researchers theorize that previous exposure to other coronaviruses (e.g., those producing many of the common colds frequently acquired by children) may confer some partial resistance to SARS-CoV-2.^47^^,^^52^^,^^54^ Interestingly, when blood samples collected before fall 2019 (i.e., before people began getting infected) were analyzed, about half the people studied appeared to already have some protective T-cell immunity to the new virus, resulting from past exposure to other coronaviruses.^60^ Importantly, young people also produce smaller amount of the aforementioned ACE-2 protein, a critical nasal cell entry point for both SARS viruses.^19^^,^^47^^,^^52^

Growing evidence suggests that children are less likely to become infected. Even if they become infected, they are less contagious than adults.

Moreover, the evidence suggests that even when children do become infected, they are probably considerably less contagious than adults.^46^^,^^53^^–^^58^^,^^62^ A recent German study found viral loads in infected children at levels comparable to adults. However, the number of children studied was very small and other methodological concerns have been raised. More importantly, although for some pathogens (such as HIV) viral load is highly associated with infectivity, the implications of viral load for COVID-19 clinical progression and contagiousness remain unclear.^53^^,^^54^^,^^61^ Because the many asymptomatic youth infected with COVID-19 are not coughing or sneezing, they emit far fewer infectious droplets. And remarkably, contact tracing studies conducted in China, Iceland, Netherlands, and United Kingdom have failed to identify a single case of child-to-adult infection of thousands of transmission events analyzed.^15^^,^^36^^,^^52^^–^^57^ A review of household transmission studies from several Asian countries concluded that less than 10% of household clusters involved a child index case,^62^ and a analysis of different COVID-19 interventions in the United States found no evidence for the impact of school closures.^63^

It should be noted that some of these data probably underestimate children’s actual contagiousness, as they were collected after lockdowns and other mitigation measures had been implemented. However, the striking findings from the contact tracing studies in particular, as well as the evidently significant biological differences between COVID-19 and other respiratory pathogens, suggest that children are not major sources of infection, especially as compared to the common cold strains of coronaviruses, for example.

Even without the substantial amount of data that emerged subsequently (which presumably would have reduced the predicted impact of school closures), in March 2020, modelers from the Imperial College of London estimated that closing schools might prevent only 2%–4% of premature deaths in the United Kingdom (i.e., predominantly of older adults with predisposing conditions such as chronic diseases, obesity, and smoking, who could become directly or indirectly infected from schoolchildren.)^64^ In contrast, the modelers estimated that 17%–21% of total deaths can be prevented from self-quarantining at home.

In Denmark, Norway, and New Zealand, where schools reopened in April 2020, the numbers of new COVID-19 cases have continued to fall, similar to trends in Finland, France, Germany, Netherlands, and Vietnam, where schools all reopened in mid-May or earlier (though cases have increased in Madagascar, but perhaps not mainly due to reopening schools). It will, of course, be vitally important to implement adequate testing and safety measures for teachers and other school employees^68^^,^^69^ and to closely monitor the data as schools also begin reopening in Australia, Israel, Japan, and elsewhere (even as some U.S. school districts and colleges have announced that fall 2020 instruction will be conducted strictly online). (In Switzerland, health authorities also announced permission for grandparents to hug their young grandchildren.^70^)

Certainly, as decisions are made regarding the reopening of schools, it must be taken into account that school closures have been depriving over a billion students worldwide of essential classroom learning, vital social connections, and physical activity. In addition, socioeconomic disparities are increasingly exacerbated, as some families have the technological, parental academic assistance, and other resources to enhance online learning, while less privileged children fall further behind.^54^^,^^55^^,^^58^^,^^71^ Other huge consequences of school closures include documented surges in child abuse; hunger from missed subsidized meals; and greater anxiety, depression and isolation, which often are most acutely experienced by students with autism, Down syndrome, attention-deficit/hyperactivity disorder and other special needs challenges.^71^^–^^80^

## ONE ALTERNATIVE TO LOCKDOWN: MOVING TOWARD HERD IMMUNITY?

Although many experts continue to believe that stay-in-place measures are needed to flatten the curve, others have proposed a Phase 2 alternative—instead of attempting to prevent any new infections—of essentially allowing younger and healthier people to gradually return to work and school, based on a herd-immunity strategy.^43^^,^^44^^,^^81^^,^^82^ Although many of them could eventually become infected, most individuals would be expected to experience relatively mild to moderate symptoms and, ideally after self-quarantining, would effectively be “naturally vaccinated” (i.e., they would presumably no longer be contagious, for perhaps a year or more). Such an approach assumes, of course, that reinfection is uncommon, which—although most experts believe is quite probably the case—remains unconfirmed.^83^^,^^84^ Note that if previous infection does not confer immunity, it may prove very difficult to develop a vaccine that does so.

This sort of herd-immunity approach could be strongly enhanced by large-scale antibody testing to identify previous infection, as China, Germany, Spain, United Kingdom, and some U.S. locales have begun to implement.^85^^,^^86^ Crucially, we must determine how best to isolate or otherwise protect the most vulnerable populations from infection—certainly no easy task. If it were to be the case, as previously discussed, that elderly but otherwise healthy people are not actually at considerably greater risk of severe illness or death, then clearly this would make the challenge somewhat less daunting. However, the evidence is not yet sufficient to base policy on this still-hypothetical possibility.

Crucially, we must determine how best to isolate or otherwise protect the most vulnerable populations from infection—certainly no easy task.

School closures have deprived over a billion students of academic progress, social connections, physical activity, and subsidized meals.

If previous infection does not provide immunity, vaccines are also unlikely to work.

Although obviously far from ideal, something akin to such an alternative approach may emerge (including perhaps in some lower-income regions) as one of the least terrible, more realistic longer-term alternatives, until a vaccine is available. Interest in such strategies is intensified by the potential for a resurgence of infections once containment measures are eased, including a possible second wave in late 2020 and early 2021. Outcomes will need to be rigorously assessed in places like Sweden, where despite most businesses and schools having stayed open, COVID-19 deaths have been declining, though not as sharply as in most other European countries.^87^

## WHAT CAN WE LEARN FROM PLACES THAT DID NOT IMPOSE A FULL LOCKDOWN?

Ongoing attention has focused on Sweden’s per-capita death rate being much higher than in other Scandinavian countries. However, a crucial difference is that in Sweden most reported cases (not only deaths) have occurred heavily among the elderly,^20^ particularly those residing in long-term care homes—similarly to the situation in Belgium, France, Italy, Netherlands, Spain, and the United Kingdom.^50^^,^^87^^,^^88^ Those countries (and, for example, the New York/New Jersey area) all have higher reported death rates than Sweden, despite tightly locking down since at least late March 2020. That Sweden’s COVID-19 mortality is lower than in those European countries becomes even more evident if comparing via excess mortality (current deaths compared to typical levels in preceding years) instead of relying on official mortality data. Belgium and Sweden are 2 European countries that appear to have maintained near completely accurate data on COVID-19 mortality.^89^ Thus, although official statistics would suggest, for example, that Belgium’s death rate is not only the world’s highest but is about double that of neighboring Netherlands’, if instead the comparison is based upon excess mortality, the 2 countries’ actual death rates appear much more similar.^89^ (It could, therefore, be mistaken to conclude that Belgium's mitigation efforts have necessarily been inferior to the Netherlands’ since the more pertinent explanation may simply involve an issue of data reporting quality.) Very importantly, all of the aforementioned places, including Sweden, have failed to implement adequate control measures in elderly residences.^50^^,^^87^^,^^88^ Whereas in Denmark and Norway, similar to the situation in Germany, Japan, and South Korea, a much larger proportion of infections has for some reason occurred in relatively younger people,^20^ consequently resulting in considerably lower COVID-19 death rates.

Apparently also very salient, if rarely mentioned, is that recent immigrants in Sweden have suffered disproportionally far greater infection and mortality rates, reportedly due in part to insufficiently targeted prevention campaigns.^90^^,^^91^ By one estimate, perhaps 40% of all COVID-19 deaths in the capital city, Stockholm, have been solely among Somali refugees (who comprise a minority of foreign-born immigrants in the city, after Iraqis, Syrians, and Afghans, in that order).^90^ Non-European residents also comprise—differently than the case elsewhere in Scandinavia—the majority of the country’s nursing home employees.^90^ Although certainly understandable, the possible fear by the Swedish government of a xenophobic or Islamophobic backlash may, however, have resulted in grave public health consequences, reminiscent of prevention campaigns during the earlier AIDS years that shifted attention, also understandably but similarly deleteriously, away from those at highest risk to avoid homophobia and discrimination against marginalized groups.^1^^,^^3^

It also appears noteworthy that in the 5 U.S. states that never imposed stricter isolation measures,^92^ observable increases in new cases have not occurred, as compared to demographically and otherwise similar neighboring rural states that implemented tight lockdowns.^93^ This observation is consistent with the fact that modeling predictions in late April 2020 of a sharp uptick of death across the United States,^94^ as many (largely rural) states began reopening, turned out to be considerably overdrawn.^93^^,^^95^

A key implication of the experience from the 5 non-lockdown U.S. states and Sweden is not that death rates in those places have been *lower* than elsewhere, but if outcomes generally have not been *worse*, this suggests that similar results may be achieved at a less drastic economic and societal cost. (In the case of Sweden, a fairer comparison would be to more epidemiologically similar European countries, rather than utilizing the “ecological fallacy” of comparing its experience only to the other Scandinavian nations.) In any case, the urge to apply an either/or, one-size-fits-all approach, which also hampered the response to AIDS and some other past health crises,^1^^,^^2^^,^^4^^,^^5^ should be questioned, including in lower- and middle-income world regions.^93^^,^^96^^–^^100^

The urge to apply an either/or, one-size-fits-all approach should be resisted.

## UNINTENDED CONSEQUENCES OF THE GLOBAL LOCKDOWN COULD BE MASSIVE

It is crucial that an evidence-based and transparent debate underpin decisions,^41^ obviously taking into consideration the unprecedented consequences of financial collapse and lost income resulting from a prolonged economic shutdown,^9^^,^^44^^,^^82^^,^^99^^–^^105^ as most painfully experienced among socioeconomically disadvantaged populations.^106^ Such disruptions are being felt most dangerously in the lowest-income regions of sub-Saharan Africa and South Asia, where the prospect looms for unintended consequences of harrowing proportions.^73^^,^^96^^–^^102^^,^^105^^,^^107^ These include potentially vast increases in deaths from malaria, tuberculosis, measles, polio, diarrheal and other diseases, and malnutrition, as vaccination, maternal and child health, family planning, and other basic services are suspended due to lockdowns or are deprioritized while health efforts increasingly focus on COVID-19.^71^^,^^96^^–^^99^^,^^108^^–^^113^ Considering that young children are likely to be particularly impacted, this would represent an even greater magnitude of devastation if measured in terms of years-of-life-lost, and not only via crude mortality numbers.

The catastrophic number of deaths directly resulting from COVID-19—which eventually may eclipse the estimated 1 million from the “Hong Kong” flu in 1968–1969,^114^ (when the world’s population was less than half of today’s)—along with the many who could suffer long-term sequelae,^115^^,^^116^ must be considered alongside the increased mortality and compromised outcomes for the numerous persons suffering from non-COVID-19-related cardiac arrest, stroke, appendicitis, and other urgent conditions who have been denied medical attention or have delayed treatment for fear of seeking hospital care.^38^^,^^117^^–^^119^

Moreover, job losses and mass school closures from the lockdowns are intensifying socioeconomic disparities, including potentially dooming hundreds of millions of children to long-term educational, psychosocial, and vocational disadvantages.^54^^,^^55^^,^^58^^,^^64^^,^^71^^,^^75^^,^^78^^,^^99^ Policy makers, such as the WHO, foreign donors, and local governments, appear to be making enormously consequential decisions without fully taking into account some key demographic as well as potentially significant climate^31^^–^^33^^,^^120^ and childhood vaccine-related^121^^,^^123^ differences between lower-income regions (characterized typically by more rural populations and an age pyramid dominated by young people) and Europe and North America (more urban, older, and often more obese populations, thus probably much more vulnerable to COVID-19 mortality).^96^^–^^99^^,^^124^^,^^125^

Furthermore, it is critical to consider the consequences of remaining inside (often cramped) living quarters for extended durations, including reported increases in domestic violence^126^^–^^128^ and child abuse,^72^^–^^74^^,^^127^ as well as other physical and mental health issues related to chronic diseases^30^; obesity^79^; social isolation^10^; anxiety, depression, and suicide^10^^,^^30^^,^^39^^,^^75^^–^^78^; obsessive-compulsive disorder^129^; poisoning from overuse of toxic cleaning products^10^; and autism, attention-deficit/hyperactivity disorder, and other developmental challenges.^80^ As has occasionally occurred with other health crises such as HIV/AIDS,^1^^,^^6^ we must not lose sight of the bigger picture. It is sadly possible, especially in the lowest-income regions, that the remedy could be worse—perhaps tragically even far worse—than the disease itself.

It may be that advanced age alone, in the absence of preexisting conditions, is less of an independent risk factor than has been assumed.

It is noteworthy that in the 5 U.S. states that never imposed stricter isolation measures, observable increases in new cases have not occurred.

## Notes

**First published online:** June 17, 2020
