# Corrigendum: Halperin DT. Coping With COVID-19: Learning From Past Pandemics to Avoid Pitfalls and Panic

**DOI:** 10.9745/GHSP-D-20-00340

**Published:** 2020-09-30

See updated article

In the article “Coping with COVID-19: learning from past pandemics to avoid pitfalls and panic” by Daniel T. Halperin, which appeared in the June 2020 issue (Volume 8, Issue 2), on page 1, the fourth sentence under the heading “Confusion and Panic Returns With This Pandemic,” was changed to “Under such circumstances, fear is understandable and can help motivate behavior change. However, when fear becomes irrational or leads to panic, it often results in poor decision making and other unintended consequences.” to avoid the incorrect implication that irrational fear can have a positive effect on motivating behavior change.

The article has been corrected accordingly.

